# Pyrometric-Based Melt Pool Monitoring Study of CuCr1Zr Processed Using L-PBF

**DOI:** 10.3390/ma13204626

**Published:** 2020-10-16

**Authors:** Katia Artzt, Martin Siggel, Jan Kleinert, Joerg Riccius, Guillermo Requena, Jan Haubrich

**Affiliations:** 1 Institute of Materials Research, German Aerospace Center (DLR e.V.; Deutsches Zentrum für Luft-und Raumfahrt), Linder Höhe, D-51147 Cologne, Germany; guillermo.requena@dlr.de (G.R.); jan.haubrich@dlr.de (J.H.); 2 Institute of Software Technology, German Aerospace Center (DLR e.V.; Deutsches Zentrum für Luft-und Raumfahrt), Linder Höhe, D-51147 Cologne, Germany; martin.siggel@dlr.de (M.S.); jan.kleinert@dlr.de (J.K.); 3 Institute of Space Propulsion, German Aerospace Center (DLR e.V.; Deutsches Zentrum für Luft-und Raumfahrt), Im Langen Grund, D-74239 Hardthausen, Germany; joerg.riccius@dlr.de; 4 Metallic Structures and Materials Systems for Aerospace Engineering, RWTH Aachen University, D-52062 Aachen, Germany

**Keywords:** laser powder bed fusion, selective laser melting, process monitoring, porosity, copper alloy

## Abstract

The potential of in situ melt pool monitoring (MPM) for parameter development and furthering the process understanding in Laser Powder Bed Fusion (LPBF) of CuCr1Zr was investigated. Commercial MPM systems are currently being developed as a quality monitoring tool with the aim of detecting faulty parts already in the build process and, thus, reducing costs in LPBF. A detailed analysis of coupon specimens allowed two processing windows to be established for a suitably dense material at layer thicknesses of 30 µm and 50 µm, which were subsequently evaluated with two complex thermomechanical-fatigue (TMF) panels. Variations due to the location on the build platform were taken into account for the parameter development. Importantly, integrally averaged MPM intensities showed no direct correlation with total porosities, while the robustness of the melting process, impacted strongly by balling, affected the scattering of the MPM response and can thus be assessed. However, the MPM results, similar to material properties such as porosity, cannot be directly transferred from coupon specimens to components due to the influence of the local part geometry and heat transport on the build platform. Different MPM intensity ranges are obtained on cuboids and TMF panels despite similar LPBF parameters. Nonetheless, besides identifying LPBF parameter windows with a stable process, MPM allowed the successful detection of individual defects on the surface and in the bulk of the large demonstrators and appears to be a suitable tool for quality monitoring during fabrication and non-destructive evaluation of the LPBF process.

## 1. Introduction

Copper alloys such as CuCr1Zr are used for the inner lining of rocket engine combustion chambers due to their high thermal conductivity and strength. The conventional manufacturing of these chambers consists of several time-consuming and costly steps [1,2]. Additive manufacturing (AM) shows good prospects for reducing lead time and costs while enabling new regenerative cooling concepts.

Copper alloys have been processed by electron beam melting (EBM) [3,4], in which larger powder sizes and high layer thicknesses (e.g., Reference [5]) commonly result in structures coarser than those achieved from laser powder bed fusion (LPBF). Therefore, the LPBF of copper alloys is being explored, leading to major challenges due to the high thermal conductivity of the powder, poor absorption of laser wavelengths in the range of ≈1070 nm found in standard LPBF machines, and balling (e.g., Reference [6,7]). As a consequence, densities for LPBF copper alloys achieved with common 200–400 W lasers are inferior to those obtained using EBM; the latter being in the range of 93.7% for Cu-4Sn [8] to 97.9% for Cu-Cr-Zr-Ti [9] and from 97.65% [10] to 99.5% [11] or even 99.8% [12,13] for CuCr1Zr. On LPBF machines with a non-standard high-powered laser, even pure copper powder can be processed: e.g., a density of 96.6% was achieved using an 800 W laser power [14].

To assure part quality in AM, non-destructive testing (NDT) methods such as computer tomography are often used after fabrication. However, as this type of ex situ testing is expensive, increasing efforts are focused toward in situ NDT methods such as, e.g., MPM. If defects can be detected during the fabrication of a part, the process can be stopped early, avoiding additional costs. Furthermore, in situ monitoring may potentially enhance the understanding of the process and enable better reproducibility and part quality. In the long term, a feedback-control system will be implemented using in situ NDT methods to enable “self-optimization of the manufacturing process” [15]. The first implementations of a feedback control that enabled the improvement of the surface quality of overhang structures [16] have already been reported.

A summary of in situ methods is provided in several reviews (e.g., References [17,18,19,20,21]). Most sensor techniques are related to either optical (e.g., References [16,22,23,24,25,26,27,28,29,30,31]) or acoustic methods (e.g., References [32,33,34,35,36]), which were often originally applied to laser welding [37]. Other methods such as low coherence interferometry [38,39], electrical methods (e.g., capacitance monitoring) [37], X-Ray backscatter imaging [40], and eddy current measurements [41] have been investigated.

Optical methods are already commercially available (e.g., References [42,43,44,45]). They are often divided into measurements with a camera (spatially resolved sensor) or with pyrometer/photodiodes (spatially integrated sensors). Combinations of both systems also exist (e.g., Reference [24]).

Pyrometer signals depend on the process parameters (powder thickness, hatch distance, and scanning velocity) [25] and correlations between photodiode signals and total porosity were first discussed in two recent studies [22,46]. Other authors using a custom developed monitoring system concluded, e.g., that in the LPBF of a 18Ni300 maraging steel thermal emission in the visible range “could be linked to the porosity” [30], and that the stability of the melt pool size could be assessed with a near-infrared detector [47].

Mapping of melt pool data on the build-plane can reveal the typical phenomenon of overheating of “overhang” structures above the powder [27]. The overheating, as visible in the MPM data, influences the microstructure formation, which was demonstrated for a Ti-6Al-4V impeller [48]. A manual analysis of melt pool fluctuations provided an inverse relationship between the plastic elongation of Ti-6Al-4V and the total volume of 3D “melt pool events” [28]. Automating the detection of melt pool events with an algorithm allowed individual pores with a volume above ≈0.001 mm^3^ (pore diameter ≈ 160 µm) to be identified, the results of which were verified by ex situ CT measurements [29].

Pyrometer measurements are commonly presented using an arbitrary unit instead of absolute temperature. For precise temperature determination, on the one hand, a complicated calibration would be necessary; on the other hand, the pyrometer field of view could be too large for the melt pool geometry, thus underestimating the temperature at the hottest location [25]. The pyrometer field of view (on-axis) used, for example, in Reference [25], of approximately 560 µm was considerably larger than the laser spot diameter of 70 µm. Ideally, this correlation should be vice versa, i.e., a larger temperature zone than the pyrometer measurement area [25]. One of the few reports providing absolute melt pool temperatures from pyrometer measurements can be found in Reference [23], where a calibration with a W lamp was employed to determine the temperature.

As the monitoring sampling zone is in most monitoring systems larger than the melt pool itself, different factors contribute to the intensity of the photodiode signal: besides the temperature of the melt pool itself, its size has also been found to be of considerable importance [28]. Furthermore, undesired influences such as balling [49], the temperature of the spatter generated by the laser, the vapor plume, and other hot spots within the observation zone can also affect the photodiode response [28].

In this work, LPBF of CuCr1Zr with a commonly-used 400 W fiber laser is investigated, which is particularly challenging because complete melting and high material densities >99% are difficult to achieve at economically attractive build-up rates at a wavelength of 1070 nm due to the high reflectivity of the material. A parameter window study is conducted at two layer thicknesses (30 µm and 50 µm) analyzing the porosity of cuboids with >100 process parameter combinations. Importantly, the role of part positioning on the base plate, which is often neglected in LPBF studies, has also been studied. Finally, a test build of large thermo-mechanical fatigue specimens with a build height of 250 mm is performed to investigate the transferability to larger components and the effects of local geometries. All builds have been monitored in situ with a pyrometric melt pool monitoring tool that shows the inhomogeneity of the thermal responses from the different cuboids, which correlates with inhomogeneous porosity. Moreover, thermal emissions from MPM provide insights into the process robustness and on the effects that local geometries in complex components exert on heat dissipation, leading to spatially varying porosity. Therefore, this study shows that besides being able to detect single, large manufacturing defects, MPM may be a suitable tool to study or even predict and adjust local material properties.

## 2. Materials and Methods 

### 2.1. Material and Processing

Water-atomized CuCr1Zr powder (0.9 wt.% Cr, 0.12 wt.% Zr; d_10_ = 21 µm, d_50_ = 33 µm, d_90_ = 44 µm) was obtained from TLS Technik GmbH & Co., Bitterfeld, Germany. Specimens were manufactured in four separate builds (Supplementary, Figure S1) using 2 mm high block supports on Ti-6Al-4V-baseplates. A standard SLM 280^HL^ machine (with a YPG 400 W fiber laser with a wavelength of 1070 nm and a 3D-scan optic; at 400 W nominal output a beam diameter of ca. 90 µm was determined from laser beam profiling) developed by SLM Solutions AG, Luebeck, Germany, was used. No baseplate heating was applied, and the manufacturing was carried out in an Ar atmosphere. 

Two types of samples were produced:

(a) For the material density (porosity) optimization and process window determination, 160 cuboids (10 × 10 × 12 mm^3^) were produced using 114 different processing parameters with hatch distances h from 80 µm to 180 µm, scan velocities v from 200 to 1000 mm/s, two different laser powers P (350 W, 400 W), and two different layer thicknesses t (30 µm and 50 µm). No separate contour strategy was used.
(b) In order to investigate the influence of upscaling and geometry, larger and geometrically challenging thermo-mechanical-fatigue (TMF) test-panels and segments thereof were manufactured with two-parameter combinations determined from the porosity optimization (t = 50 µm, P = 400 W, h = 120 µm and two different velocities v = 300 mm/s and 500 mm/s).

For simplification and trend analysis, we will refer to a convoluted laser parameter descriptor, i.e., the volume energy density:

(1) $$E_{v}=\frac{P}{h\cdot t\cdot v}\;[J/{\mathrm{mm}}^{3}].$$

### 2.2. Experimental Methods

Light optical microscopy (LOM) and geometry analysis were conducted with a digital microscope Keyence VHX-1000 (using focus variation; VH-Z20R objective; image stack ∆z = 20 µm; Keyence GmBH, Neu-Isenburg, Germany). The LOM of polished sections was performed with a Carl Zeiss Axio Observer microscope (Zeiss AG, Oberkochen, Germany). The sample preparation consisted of iterative steps of grinding with abrasive paper starting at grit 500 up and followed by up to 2400 grit and successive polishing with 3 µm diamond suspension, 1 µm, 0.25 µm, and 0.04 µm for 5, 2, 2, and 5 min each at ≈20 N. The samples were ultrasonically cleaned in ethanol between the different steps.

The porosity was determined by the Archimedes method employing a KERN precision balance ABT 120-5DM (Kern & Sohn GmbH, Balingen, Germany) and deionized water at room temperature. The theoretical density of CuCr1Zr alloy amounts to 8.91 g/cm^3^ [50]. The porosity analysis was carried out on the basis of ISO 3369:2006 [51].

### 2.3. MPM

**Figure 1:**
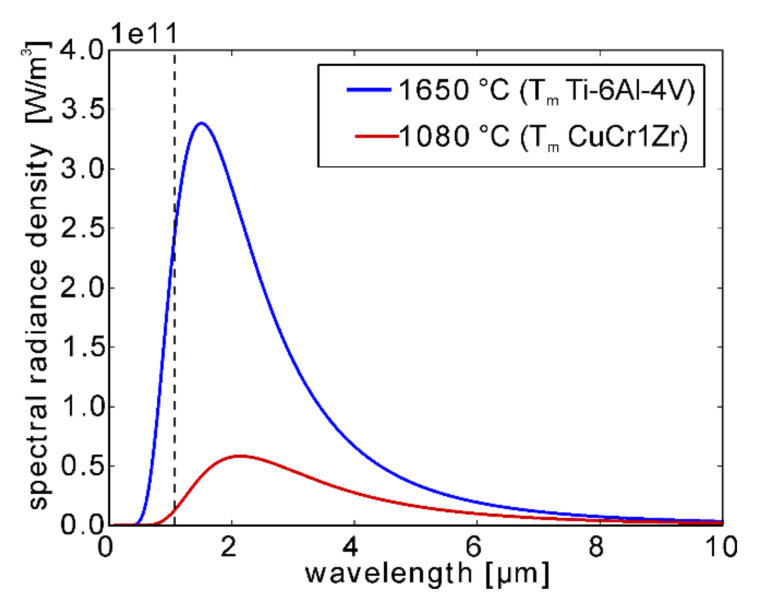
Spectral radiance of an ideal black body according to Planck’s law for two different temperatures corresponding to the melting temperatures of CuCr1Zr and Ti-6Al-4V. For reference, the wavelength of the laser is marked by a black dotted line. Significantly lower MPM intensities can be expected for CuCr1Zr due to the lower melting point in the black body approximation.

An MPM module provided by SLM Solutions AG (Luebeck, Germany) [41,43] was used. This on-axis monitoring system consists of two photodiodes with different sensitive wavelength regions. Only an arbitrary unit for the recorded intensities will be presented. The response of the MPM, i.e., the number of photons recorded by the photodiodes, is influenced by the melt temperature as well as the melt pool size. Owing to the low melting temperature of CuCr1Zr (solidus temperature 1070 ℃ and liquidus temperature 1080 ℃ [50]) compared to the melting temperature of e.g., Ti-6Al-4V (1670 ℃ [52,53]), which is one of the most used materials for AM, only comparably low absolute intensities are expected to be monitored for CuCr1Zr according to Planck’s law (Figure 1).

The intensities of both photodiodes and the lateral coordinates are saved by the MPM module in each layer, which enables a detailed analysis of the MPM data along each vector and a layer-wise mapping of the MPM data on pre-selected grids. The acquisition rate was selected to be 10 µs for the first three build jobs and reduced to 20 µs for the longest build job (Supplementary Figure S1). Thus, MPM data volumes of 186 GB (build I, c.f. Figure S1), 73 GB (build II), 500 GB (build III), and 335 GB (build IV) were acquired during the individual LPBF jobs.

The data were analyzed using a custom Python program for calculating mean values and standard deviations and with a software tool developed at the DLR Institute of Software Technology for visualization and data remapping of single layers. The data exported with this tool was subsequently analyzed three-dimensionally with the AVIZO^®^ software package (version 2020.1, Thermo Fisher Scientific, Waltham, MA, USA). Only data points with an active laser condition were taken into account (i.e., no “dark” points).

The data sets were reduced in size to handle the high amount of data recorded by the MPM system:

1. For cuboids: when calculating the mean values of an MPM intensity (or the quotient) per layer, python scripts that take a data point every 200 µs into account were devised. This amounts to 2 × 10^6^ non-zero values for the total built platform per layer, or ≈25,000 data points for each specimen per layer, respectively. The 3D visualization was carried out with a DLR-software in conjunction with AVIZO. From the build with t = 30 µm, data points were read every 50 µs and averaged onto a grid with a voxel size of 0.09 × 0.09 × 0.03 mm^3^. From the build with t = 50 µm, data points were read every 50 µs and averaged onto a grid with a voxel size of 0.09 × 0.09 × 0.05 mm^3^.
2. For the build with segments of TMF-panels: data points were read and averaged every 50 µs onto a grid with a voxel size of 0.15 × 0.15 × 0.15 mm^3^.
3. For the build with full TMF-panels: data points were read and averaged every 100 µs onto a grid with a voxel size of 0.15 × 0.15 × 0.15 mm^3^.

## 3. Results and Discussion

### 3.1. Part I: Cuboids

#### 3.1.1. Density Analysis with LOM and Archimedes Method

**Figure 2:**
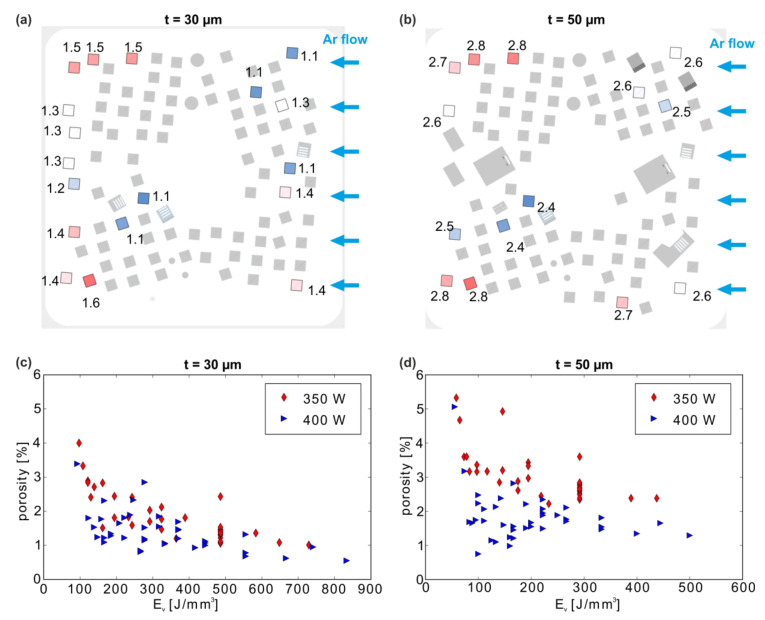
(**a**), (**b**): Reproducibility study: the distribution of nominally identical specimens (P = 350 W, v = 300 mm/s, h = 80 µm) on the base plate for two build jobs with different layer thicknesses t with resulting porosities, rounded to 1 decimal [%] (red color indicates higher, blue lower porosity). (**c**), (**d**) Porosity of all tested specimens from both build jobs vs. the volume energy density.

The first step of the study consisted of the determination of two process windows from small cuboids (10 × 10 × 12 mm^3^) manufactured with 114 different process parameter combinations at a chosen layer thickness of 30 and 50 µm. Archimedes density measurements were conducted for all cuboid specimens with an uncertainty estimated at 0.4% (calculated by error propagation; each sample was measured three times) in order to determine suitable LPBF parameter combinations for dense materials as well as studying the influence of the build platform positioning. The literature generally indicates that an average laser power of 400 W might not be sufficient for manufacturing highly dense material (>99%) at least at higher build-up rates using layer thicknesses above 20 µm (e.g., Reference [54]). However, Uhlmann et al. reported a total porosity of 0.5% in CuCr1Zr for a low laser power of 350 W [11] at a high layer thickness of 50 µm, which we thus included in our parameter variations. With the best LPBF process parameters from the latter study (P = 350 W, v = 300 mm/s, h = 80 µm, t = 50 µm), 14 nominally identical specimens were manufactured at different positions on the base plate for testing reproducibility and base plate positioning effects (Figure 2a,b). 

The porosities resulting after cuboid manufacturing with different parameters varied between 1.1–1.6% ± 0.4% (t = 30 µm; E_v_ ≈ 500 J/mm^3^) and 2.4–2.9% ± 0.4% (t = 50 µm; E_v_ ≈ 300 J/mm^3^). These values are higher than those reported in Reference [11], demonstrating that porosity cannot be transferred easily between LPBF machines. A number of factors, including the use of slightly different setups (i.e., an SLM 250^HL^ in Reference [11]), build temperatures (200 ℃), the quality of the gas-atomized CuCr1Zr, or ad-/absorbents such as, e.g., H_2_O or H_2_ [55], may influence the material quality. Importantly, the former authors used laboratory CT with a comparatively low spatial resolution for analysis, which can lead to an underestimation of porosity. In contrast, Guan et al. also reported a higher porosity of 2.35% after LPBF of gas atomized CuCr1Zr with the parameters 425 W, 350 mm/s, and 90 µm hatch distance at a layer thickness of 20 µm using an EOS M250 setup [10]. This latter result is more in line with our data, although the lower layer thickness should usually even at relatively low laser powers of ≈400 W lead to much higher volume energy densities and fully molten, denser the material. Using the same layer thickness, Wallis et al. showed that this increased volume energy density was required for obtaining low porosities down to 0.2% (single scan strategy) or even 0.01% (remelting scan strategy) with a 400 W laser [12] (the porosity was determined with light optical microscopy from microsections in the latter study).

A closer look at the scattering of the data points for samples manufactured with nominally identical LPBF parameters reveals a correlation between the sample’s position on the baseplate and the resulting porosity: in the center of the baseplate (Figure 2a,b) slightly lower porosities are obtained than in the upper and lower areas of the build space. The overall spread amounts to ≈0.5% for both layer thicknesses. One potential reason for this observation might stem from an inhomogeneous Argon flow in the build space (flow in Figure 2a,b from right to left), which is known to result in an inferior process atmosphere [56,57] and has been related to defect formation [29]. 

This position effect must also be considered when deriving general tendencies from the measured porosity between samples of varying parameters. Hence, porosity results obtained from a single specimen should always be taken with care, particularly when very high material densities are obtained. 

For the detailed analysis of the parameter influence, a combined representation (Figure 2c,d) is chosen. It illustrates that the porosity of all manufactured cuboids is on average lower for a smaller layer thickness of t = 30 µm, for which it ranged from 0.5% to 4.0% (Figure 2c), and slightly higher from 0.8% to 5.4% for t = 50 µm (Figure 2d). Independent of the layer thickness, lower porosities are achieved, as expected, with the higher laser power (400 W). The much larger range of E_V_ for the LPBF parameters at thinner 30 µm layers (≈100–850 J/mm^3^ versus ≈80–500 J/mm^3^ for t = 50 µm) lead to rather comparable variations in porosity, which may indicate that the melting process is less sensitive to parameter changes for thinner layer thicknesses.

Furthermore, this convoluted representation (Figure 2c,d) shows the width of the scatter band for the single specimens discussed above (stack of red data points for 350 W at v = 500 mm/s and 300 mm/s for t = 30 µm and 50 µm, respectively). Meanwhile, for t = 30 µm, the lowest porosities are achieved at high energy densities; for t = 50 µm, low porosities can also be reached at low E_v_. Thus, even high scan velocities and hatch distances (i.e., low E_V_ = 100 J/mm^3^) result in porosities similar to those obtained at low velocities (i.e., high E_V_ = 500 J/mm^3^) for t = 50 µm. This translates directly into an economic advantage: at a build-up rate of 14 cm^3^/h, a material density comparable to that at 2.8 cm^3^/h can be obtained.

**Figure 3:**
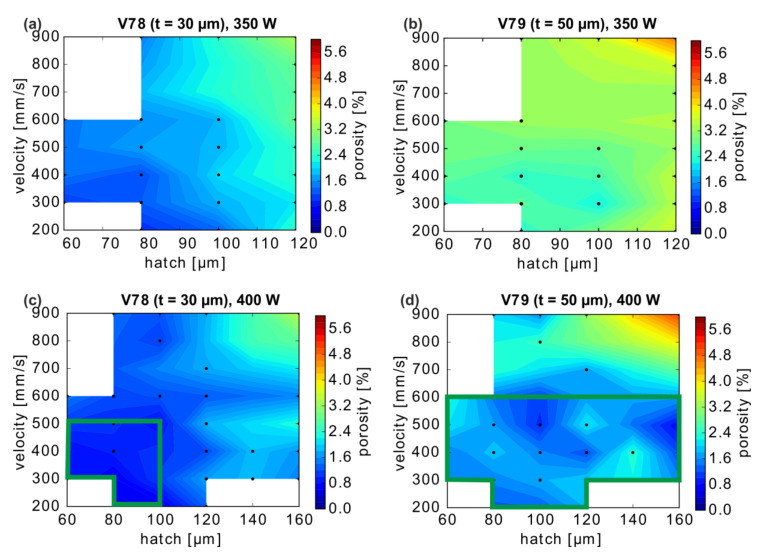
LPBF-CuCr1Zr-Porosity dependence on layer thickness t, laser power, velocity, and hatch distance (linear interpolation of porosity between data points). (**a**) and (**c**) t = 30 µm, (**b**) and (**d**) t = 50 µm, for P = 350 W and 400 W, respectively. Because not all process parameter combinations were measured (i.e., white regions), the chosen combinations are marked with black dots. The green rectangle indicates a possible process window for a given laser power of 400 W for 2 different layer thicknesses t.

Further insights on the effects of the scan velocity v and hatch parameter h are derived from the porosity maps presented in Figure 3. As expected, a combination of high scan speeds with high hatch distances generally leads to incomplete melting and an increasing porosity independent of the chosen layer thickness or laser power. This has been attributed to the decreasing overlap of the melt tracks, which decrease in width at high scan speeds [10]. For a layer thickness of 30 µm, the variation of the hatch distance in the range of 60 to 180 µm leads to significant changes in porosity, which is generally lower for a smaller h. A suitable process window with acceptable porosity is found at 400 W with 60 µm ≤ h ≤ 100 µm and 200 mm/s ≤ v ≤ 500 mm/s (minimum: 0.5% for v = 200 mm/s, h = 80 µm), marked with a green border in Figure 3.

In contrast, at a layer thickness of t = 50 µm and 400 W, the influence of hatch distances between 60 µm to 160 µm is almost negligible. A large process window at 400 W laser power and t = 50 µm can, thus, be defined with hatch and velocity combinations from 60 µm ≤ h ≤ 160 µm and ≈200 mm/s ≤ v ≤ 600 mm/s (green rectangle in Figure 3d), in which the lowest porosity of 0.8% was achieved with h = 160 µm and v = 500 mm/s. Importantly, this value is only slightly above the minimum achieved at t = 30 µm, particularly considering the intra-build scattering. Thus, a layer thickness of 50 µm appears to be optimal from a productivity perspective.

**Figure 4:**
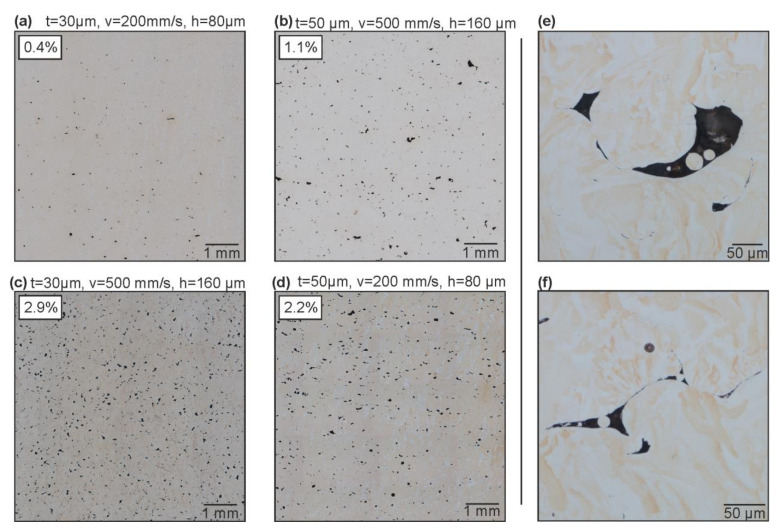
Examples of typical pore shapes obtained with P = 400 W and different layer thicknesses t = 30 µm (**a**,**d**) and t = 50 µm (**b**,**c**). LOM of specimens manufactured with P = 400 W, v = 500 mm/s, and t = 50 µm (**e**,**f**). The total porosity determined from the microsections is presented on the top left of each figure.

In order to substantiate the hypothesis of the role of balling on the bulk porosity, sections of the LPBF specimen fabricated at both layer thicknesses were analyzed with LOM (Figure 4). Polished sections were analyzed, and selected images of the samples built with parameters optimized at the specific layer thickness are shown (Figure 4a,b). Moreover, the samples built at the alternative layer thickness with the same parameters, i.e., in a non-optimal condition, are presented (Figure 4c,d). 

The typical defect shapes correspond to balling effects, with sharp edges due to a lack of fusion and containing unmolten powder (Figure 4e,f). This is likely linked to insufficient energy input, i.e., low laser power for the given hatch, velocity, and layer thickness. The balling defects can exceed the layer height in dimension, sometimes measuring in excess of 100 µm (z_max_). These dimensions are in rough agreement with those from the larger observed balling protrusions inferred from LOM surface maps of the top surfaces of the specimen (see Supplementary Material, Figures S2 and S3). The porosity as well as the pore size are on average smaller for specimens manufactured with the lower layer thickness (t = 30 µm; Figure 4a), although, when moving far away from the optimal parameters, exceptions are found, e.g., at high hatch distances of 160 µm (Figure 4c). This is in agreement with the trends from the Archimedes’ porosity measurements.

#### 3.1.2. MPM of Coupon Specimen

**Figure 5:**
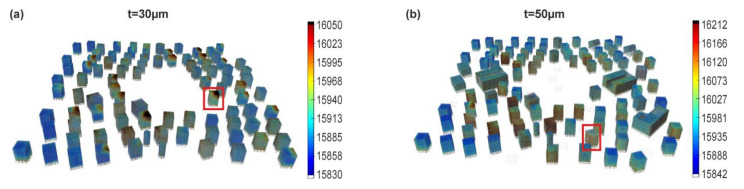
3D-visualization of area-mean intensity, I_2_, for the two builds (**a**) t = 30 µm and (**b**) t = 50 µm. Two specimens are marked with red boxes and will be discussed in detail below. Each shown cuboid measures 10 × 10 × 12 mm^3^.

In order to investigate whether the melt pool data monitoring is useful for the analysis of the porosity trends or it can be used predictively for parameter development, the MPM data is analyzed first in a layer-wise fashion. The data is then averaged for the volume of each cuboid sample of the build process. The total MPM response during the fabrication of the cuboids appeared less homogeneous within every single specimen at t = 30 µm (Figure 5a), with “hot spots” at the top right sides in contrast to t = 50 µm (Figure 5b). Hence, the changes in the MPM responses between subsequent layers were studied first.

To this purpose, area-mean intensities (Ī_2_; the bar indicates area-averaged values) and area standard deviations (σ) were calculated separately for each layer (and each photodiode) and presented as a function of the build height (one representative example is provided in Figure S4). Two transitions from the Ti-6Al-4V base plate to the supports (build height b = 0 mm) and from the supports to the cuboid bulk (2 mm) lead to increased emissions, in the latter case of which is due to the loose powder below most parts of the first “massive” layer of the bulk specimen. The powder particularly reduces the local heat conductivity (loss) and, thus, increases the melt temperature.

For analysis of all manufactured cuboids, only the layers from 3 to 14 mm build height (i.e., leaving out a distance of 1 mm from the bottom of the sample) were considered, which showed rather constant mean intensities (Figure S4a) within a small scattering band (Figure S4b). Therefore, the integrally averaged intensity can be computed and analyzed for each cuboid.

**Figure 6:**
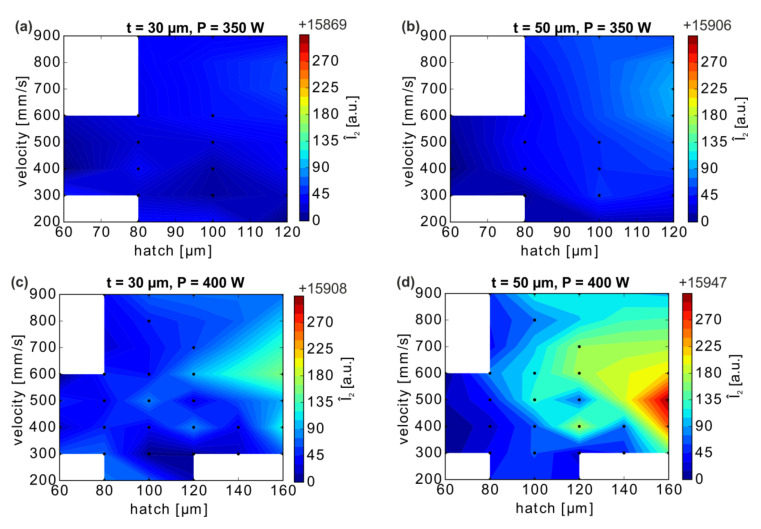
Volume-mean intensity Î_2_ as a function of the velocity and hatch distance parameters computed for the bulk of the cuboid specimen (i.e., above the supports, 3 mm ≤ b ≤ 14 mm): (**a**) and (**c**) t = 30 µm, (**b**) and (**d**) t = 50 µm, for P = 350 W and 400 W, respectively. The values between the analyzed, black marked data points were linearly interpolated.

The layer-wise mean intensities Ī_2_ were then averaged in each cuboid to the bulk mean intensities Î_2_ (the circumflex indicates volume-averaged values) and plotted against the corresponding velocities and hatch distances (Figure 6); the results illustrate very similar trends for both layer thicknesses and both laser powers. The computed bulk mean intensities Î_2_ at P = 350 W are highest for the largest chosen hatch distance h = 120 µm and velocities in the range of ≈600 mm/s ≤ v ≤ ≈800 mm/s (Figure 6a,b). Reducing h or changing v leads to a small intensity decrease in the case of a 30 µm layer thickness, although another region of increased Î_2_ can (barely) be recognized for minimal velocities and hatch values (Figure 6a). For the layer thickness of 50 µm, merely any decrease in Î_2_ outside the above-mentioned window is detected. These observations are readily transferable to the samples manufactured with a laser power P = 400 W (Figure 6c,d).

The highest MPM intensities are obtained at large hatch distances and intermediate velocities around 500 to 600 mm/s. The higher Î_2_ values at increased hatch distances result from the amount of powder available for melting and, thus, a larger melt pool size.

The regions of varying mean Î_2_ (Figure 6) can be compared to the total porosity maps of the respective cuboids (Figure 3). No direct correlation between porosity and mean MPM intensity is apparent. Parameter combinations of Î_2_ maxima and minima, as well as their trends, do not directly follow the variations of porosity of the respective specimen. Importantly, high Î_2_ values do not correlate automatically with low porosities.

**Figure 7:**
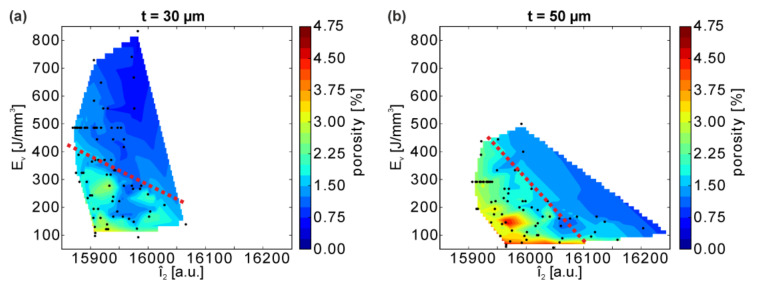
Porosity correlation with the volume energy density E_v_ and the bulk mean intensity Î_2_ for 2 different layer thicknesses. (**a**) t = 30 µm and (**b**) t = 50 µm. The black dots indicate measured data points and the regions in between are linearly interpolated.

Further insights on the process conditions and the resultant porosity can be obtained by using a combination of volume energy density E_V_ and bulk mean intensity Î_2_ (Figure 7): generally higher E_v_ and Î_2_ (i.e., toward the top right corner of each diagram) correlate to a lower porosity in the specimen.

Moreover, this representation illustrates that the degree of dependence between porosity and Î_2_ as well as porosity and E_v_ vary significantly with the powder layer thickness. By approximating an “iso-porosity-line” that roughly separates the regimes with lower and higher porosities (red dotted lines in Figure 7), a steeper slope and hence a stronger dependence between porosity and Î_2_ in the case of thicker 50 µm powder layers as compared to 30 µm is observed, indicating that variations in Î_2_ can be traced back more unambiguously to the porosity (i.e., higher Î_2_ clearly linked to lower total porosity). At t = 30 µm, the iso-porosity-line is flatter, suggesting instead that a narrow interval of Î_2_ relates to a wide range of porosities, i.e., providing only a weak correlation between porosity and Î_2_, but a strong correlation of E_V_ and porosity.

LPBF parameter combinations of identical E_V_ (e.g., the horizontal line of black dots along at E_v_ = 486 J/mm^3^ in Figure 7a, t = 30 µm) lead to different melting conditions, as evidenced by comparably strongly varying Î_2_ (i.e., from ≈15,870 to ≈15,955 a.u. corresponding to about 40% of this whole range of detected intensities) and consequently result in varying porosities. Thus, neither the total porosity nor the bulk mean MPM intensity show an unambiguous relationship as a function of E_V_, showing again that E_V_ needs to be applied carefully for a process description and that the single parameters (P, v, h, etc.) ought to be considered instead, in order to provide a thorough understanding. E_V_ generally is not an unambiguous property to correlate properties such as porosity can easily be understood from a thought experiment. Let us assume two specimens are produced at the same, constant E_V_: One specimen with a medium hatch distance and scan velocity (chosen for giving dense material), and one specimen with a very large hatch distance exceeding the melt track width (e.g., h = 1000 µm) and, correspondingly, very low velocity. In case of a very large hatch distance, only single melt tracks form with loose powder in between them. Even wider melt tracks at very low scan velocities cannot compensate the effect of the high hatch distance. As a consequence, despite E_v_ being chosen identically, the sample produced with such a high hatch distance will be highly porous in contrast to the other specimen with dense material. Therefore, E_V_ should be used carefully as a descriptor.

Similar to the sensitivity of the porosity toward parameter variations discussed above (see Section 3.1.1), the stronger correlation between E_v_ and Î_2_ for the thicker powder layer may be attributed to the larger volume of material melted before the energy dissipates into the bulk below.

**Figure 8:**
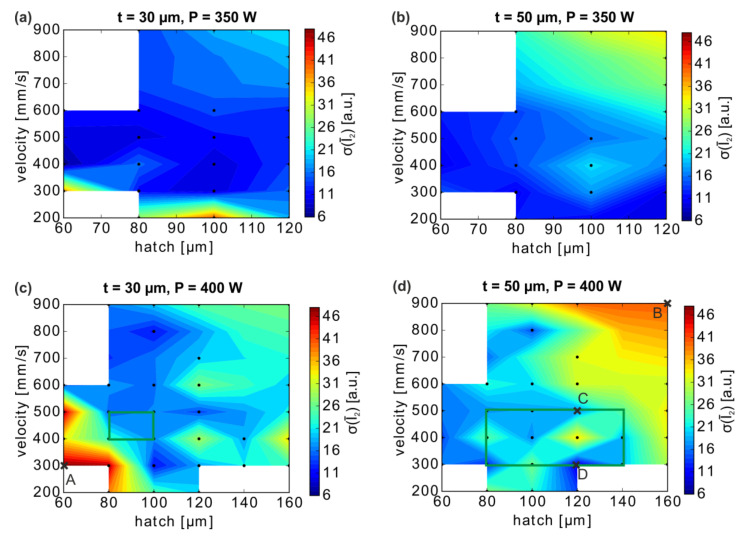
(**a**–**d**) Standard deviation σ(Ī_2_) calculated from the layer-wise mean intensities Ī_2_ (cuboids above the supports, 3 mm ≤ b ≤ 14 mm), depending on the layer thickness t and power P. A linear fitting of σ(Ī_2_) has been applied between the data points (black dots). The positions of two specific specimens are marked with crosses A and B in (**c**) and (**d**), which have a particularly high σ(Ī_2_). The boxes mark parameter windows and the crosses C and D parameter combinations selected for further component manufacturing.

The variation of the MPM intensity during the builds represents additional information on the processing conditions that is important for the selection of a processing window. Besides the volume-mean intensity Î_2_, the standard deviation of all layers σ(Ī_2_) was also analyzed for each specimen (Figure 8; again, for the volume above the supports). The standard deviation provides information on the MPM variations in a cuboid across the build height (see the inhomogeneities mentioned in Figure 8a) and, more importantly, allows assessing the overall stability of the melting process.

For the build with a layer thickness of 50 µm, the standard deviation σ(Ī_2_) increases substantially with increasing scan velocity and hatch distance. In contrast, at t = 30 µm, only a small σ(Ī_2_) increase occurs in this region and the main effect is observed toward small v and h parameters. Generally, the amplitudes of the standard deviations are somewhat larger for the smaller Ī_2_ values obtained at the smaller layer thickness of 30 µm (see Figure 6 above), which indicates a slightly higher process robustness at t = 50 µm.

The σ(Ī_2_) trends thus suggest that, at both layer thicknesses, LPBF parameter combinations of the intermediate scan, velocities, and hatch distances may provide more stable melting conditions. Seeking a compromise of low porosity and low scattering, the process windows can be narrowed down to (i) t = 30 µm: P = 400 W with 80 µm ≤ h ≤ 100 µm and 400 mm/s ≤ v ≤ 500 mm/s (lower build-up rates) and (ii) t = 50 µm: P = 400 W with 80 µm ≤ h ≤ 140 µm and 300 mm/s ≤ v ≤ 500 mm/s.

**Figure 9:**
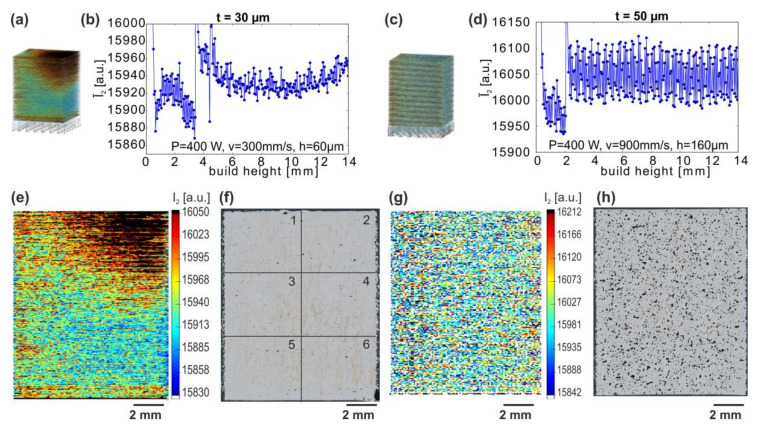
3D visualization of MPM data (Ī_2_; (**a**,**c**)), corresponding layer-averaged plots over the build height (**b**,**d**) and MPM slices (**e**,**g**) versus corresponding light optical microscopy sections (**f**,**h**) for selected cuboids which were manufactured with different process parameters (t = 30 µm, P = 400 W, v = 300 mm/s, h = 60 µm and t = 50 µm, P = 400 W, v = 900 mm/s, h = 160 µm). Both specimens (baseplate positions marked in Figure 2a,b with red boxes) show high scattering (Figure 8c,d, marked A and B). The LOM image (**f**) is divided into 6 areas for further analysis.

A layer-by-layer analysis of the Ī_2_ variations for different cuboids reveals spatial inhomogeneities (Figure 9a,c). Hence, MPM is suitable to characterize the stability or homogeneity of the melting conditions within specimens, which, as will be discussed below, lead to material inhomogeneities. The mean intensities Ī_2_ along the build height for two extreme cases of cuboids with high standard deviation (Figure 9b,d) and parameters marked by crosses A and B in Figure 8c,d show two contributions to the scattering: a spatial change of local I_2_ across the build height or sides of the cuboids, and the layer-to-layer variations of the area-mean Ī_2_.

The cuboid chosen for the thinner layer t = 30 µm (P = 400 W, h = 60 µm, v = 300 mm/s) clearly shows that the spatial distribution of I_2_ can be far from homogenous in the specimen: the upper right part and particularly the top right corner show sizably increased I_2_ values compared to the rest of a cuboid’s volume, suggesting inhomogeneous melting conditions in the volumes (Figure 9e). This causes the mean intensity Ī_2_ to shift significantly over the complete build height (2–14 mm; Figure 9b). This shift in I_2_ may also contribute to the weak correlation between E_v_, I_2_ and porosity for t = 30 µm (Figure 7a). In conjunction with small variations from layer-to-layer (at the order of ca. 40 a.u.), the shift results in an overall high standard deviation σ(Ī_2_) for the cuboid manufactured with this LPBF parameter set.

This type of spatial variation occurs predominantly at low hatch distances and scan velocities (and low layer thickness), with the resulting higher E_V_ promoting the phenomenon. They are not dependent on the build plate positioning or the inert gas flow, but can be understood from the chosen scan strategy: the vectors in each layer are generally lasered roughly from left to right (against the inert gas flow), explaining why heat may accumulate at the right side of the cuboid.

LOM cross-sections of the cuboids show that the inhomogeneous I_2_ distributions coincide with locally inhomogeneous materials (Figure 9): a porosity gradient exists in the specimen manufactured at t = 30 µm, linking higher MPM intensity to a lower total porosity yet significantly larger pores. Four LOM cross-sections were recorded and analyzed in 6 sections (three in height and two in width, 4.6 × 3.8 mm^2^ each), results see Table 1.

In the upper right segment (area 2) with the highest I_2_ values, a slightly lower porosity (0.40 ± 0.11%) was found compared to the other segments (0.59 ± 0.18%). Furthermore, half as many pores (190 ± 60) were observed in this area as compared to the rest (370 ± 40 pores). While generally half of the pores were larger than 120 ± 26 µm^2^, a higher fraction of larger pores exists in area 2: roughly 10% of all pores are larger than ≈840 ± 270 µm^2^, whereas the mean value for the remaining sections is in the range of 670 ± 130 µm^2^.

In contrast, for the t = 50 µm specimen, no distinct differences in the different segments were found. To counter these effects, an alternative scan strategy may be beneficial. Usually, the scan starts from the left side to the right, which is the opposite direction of the Argon flow, thus avoiding negative influences from spatter or smoke (e.g., Reference [58]). However, in order to promote stable process conditions in the case of CuCr1Zr at low layer thicknesses (t = 30 µm), a less constraint variation of the scan directions could be considered.

For the chosen cuboid produced at t = 50 µm, I_2_ is more homogeneous within the volume (P = 400 W, h = 160 µm, v = 900 mm/s; Figure 9c), indicating that the process and heat transfer are more stable at a higher layer thickness. No spatial variations of Ī_2_ are obtained in this case (Figure 9g). The changes of Ī_2_ from layer-to-layer, however, exhibit a sizable scatter amplitude (≈200 a.u.), which again leads to a high standard deviation σ(Ī_2_).

To summarize, the spatial distribution of I_2_ MPM response and the layer-averaged Ī_2_ allow us to assess the local melt process stability and provide indications of the local material properties (“local” porosity), whereas the integrally averaged Î_2_ provides—although not unambiguously on its own—an indication of the total porosity to be expected for a specimen. While a single integral MPM value for a specimen is not sufficient for a detailed description of its processing result, it is found to be useful in conjunction with E_V_ for the guidance of process parameter developments. Moreover, the scattering or changes of Ī_2_ between the layers of a cuboid also proves to aid the process developments as it provides hints on the process robustness for parameter sets. Combinations of low total porosity and low standard deviations σ(Ī_2_) will be chosen for part manufacturing, as discussed below.

### 3.2. Part II: Components

#### 3.2.1. Influence of Geometry and Build Strategy on Porosity

The objective of AM is the fabrication of components. However, the prior optimization of the process parameters used for components is typically performed using small bulk specimens. In such a case, the small specimen does not show the same processing behavior and thermal history like larger and complex shaped parts, as many factors, including the local geometry, wall thicknesses, orientation, supports, etc., influence the result.

**Figure 10:**
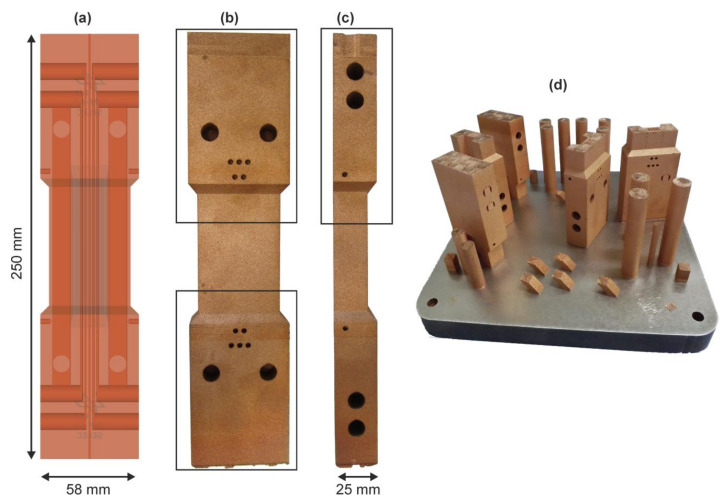
(**a**) Semi-transparent CAD model of a TMF-panel indicating the channels for internal cooling and instrumentation; (**b**) and (**c**) side views of a fabricated panel with top and bottom sections marked by boxes; (**d**) a build with geometrically difficult top and bottom sections of these panels as well as numerous other samples.

In order to investigate the transferability of the processing parameters to a more complex model component and to assess the effects of geometry, the LPBF manufacturing of large panels for thermo-mechanical-fatigue (TMF; Figure 10) tests was investigated subsequent to the coupon level studies. The panels measure 25 × 58 × 250 mm^3^ and contain several internal channels (of round and flat elongated types; Figure 10a) as well as overhanging structure features, thus providing substantial geometry challenges as compared to simple bulk forms.

For component manufacturing, two sets of process parameters were chosen within the determined process windows as identified based on the combination of low total porosity, stable processing conditions (low scattering), and high build-up rates: t = 50 µm layer thickness, P = 400 W laser power and an intermediate hatch distance of 120 µm at two different velocities v = 300 mm/s and 500 mm/s.

Initially, geometry effects were studied on segments corresponding to the upper or the lower half of the panels (109 mm out of 250 mm total height; sections marked in Figure 10b,c) with boxes and corresponding build (in Figure 10d) which were manufactured varying the build orientation, supports, and the scan velocity.

**Figure 11:**
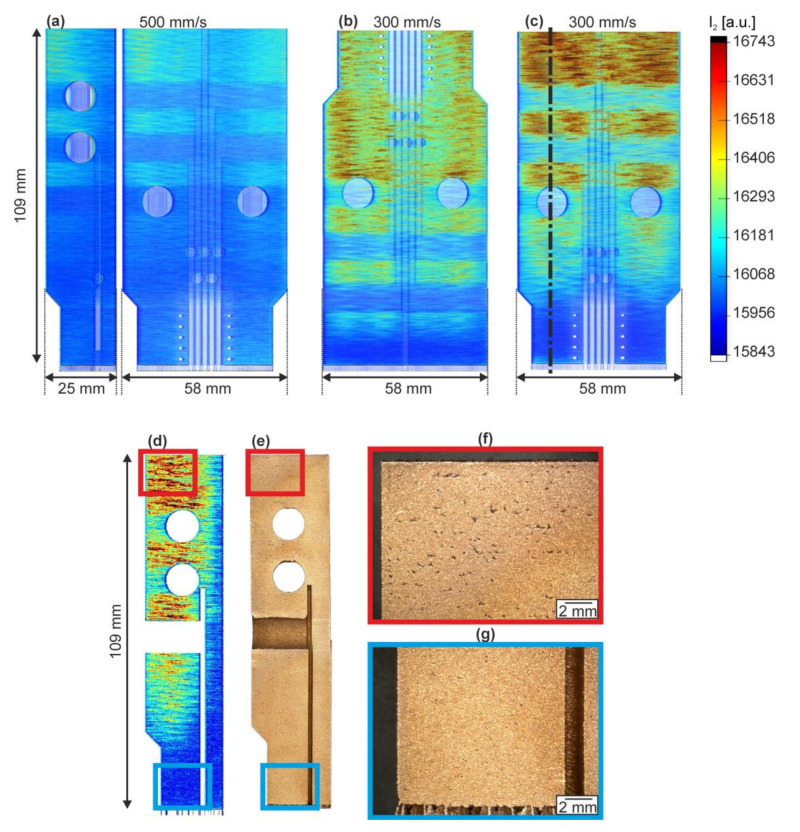
3D visualization of the MPM response I_2_ for 3 different TMF-panel segments (all: P = 400 W, t = 50 µm, h = 120 µm; visualization voxel size 0.15 × 0.15 × 0.15 mm^3^). (**a**) top end manufactured with v = 500 mm/s and supports in channel openings; (**b**) bottom end, v = 300 mm/s; (**c**) top end, v = 300 mm/s. The dimensions of the segments are 25 × 58 × 109 mm^3^. Corresponding cross-sections of I_2_ (**d**) and the manufactured segment (**e**) with markings corresponding to the LOM images show different material densities in the upper (**f**) and the bottom part (**g**).

With the transition to parts of more complex geometry, the effect of the process parameters on the intensity is more pronounced, and the intensity distribution is strongly influenced by the local geometry (Figure 11). In locations near the baseplate, the MPM intensities are generally lower, because of the fast heat transfer to the colder baseplate. Contrary to the behavior of small cuboids with corresponding LPBF parameters discussed above, which showed almost identical Ī_2_ for 300 mm/s and 500 mm/s (P = 400 W, h = 120 µm), the MPM response of I_2_ is significantly higher in the case of the panels produced with v = 300 mm/s (Figure 11b,c) compared to that with v = 500 mm/s (Figure 11a), especially with increasing part height. Hence, the MPM results from cuboids are not directly transferable to more complex parts.

Moreover, the MPM signals and thus the thermal profiles vary strongly with the internal panel geometry along their build height: the heat transfer toward the base plate is impeded by geometrical features which reduce the cross-section of the panels, such as, in particular, the large bore holes for the cooling medium connecting internally to flat channels along the height of the TMF-panel (Figure 11). These holes cause heat accumulation in the sections above, which is clearly recognized in the MPM data (Figure 11b–d). Similarly, the general reduction in the TMF specimens’ center results in a slight heat accumulation in the upper half of the panel that is evidenced by MPM. Although both the top and bottom sections shown in Figure 11b,c were manufactured similarly with v = 300 mm/s, they led to a considerably higher I_2_ in the upper part of the top section (Figure 11c).

Further LOM analysis of one of the panel’s top segments carried out on a representative plane (Figure 11c, marked line corresponding to the left region of the segment) revealed that the changing thermal history along the build height directly influenced the local material properties. Evidently, the pore sizes in the top (Figure 11f) and bottom regions (Figure 11g) of the part vary strongly following the changes in I_2_ (Figure 11d): the much higher I_2_ responses at the top showing poorer heat dissipation correlate with qualitatively large pore sizes. Hence, the spatial analysis of the I_2_ response can also provide insights or predictions of the obtained material properties for more complex parts. 

#### 3.2.2. Identification of Single Part Irregularities from MPM

Besides establishing variations in the local thermal history during the processing linked to the resulting porosity, MPM was able to resolve different types of individual yet larger defects on the surfaces and in the bulk of LPBF manufactured specimens and parts. Two irregularities shall be presented briefly.

**Figure 12:**
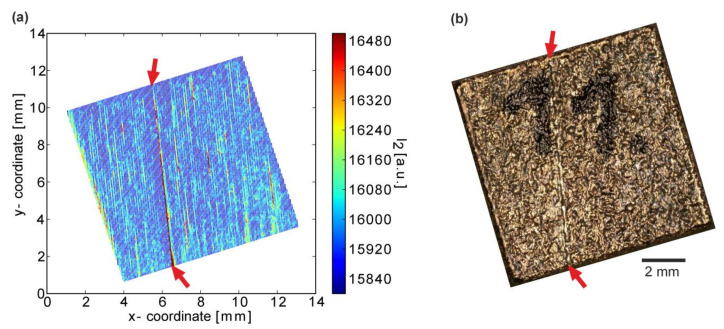
(**a**) Example for MPM data I_2_ for the last layer of a specimen (process parameters: t = 50 µm, P = 400 W, v = 400 mm/s, h = 60 µm) and (**b**) the corresponding LOM-image. A line-shaped process irregularity is indicated by the red arrows.

On the top surface of a cuboid, one pronounced but fine “hotspot line” of the width of a single melt track was detected (Figure 12a). LOM of this specimen evidences a marked trench on the otherwise typical LPBF top surface (Figure 12b). No further correspondence of this line irregularity was found in deeper layers, indicating that this event occurred only in the last layer. The line is not oriented perfectly vertically, as would, for instance, be expected for coating failure. However, while the coater may have pushed a larger agglomerated particle with a slight sideways movement and affected the powder bed, a laser instability might also account for this inhomogeneity as the direction agrees with that of the scan vectors of the last layer.

**Figure 13:**
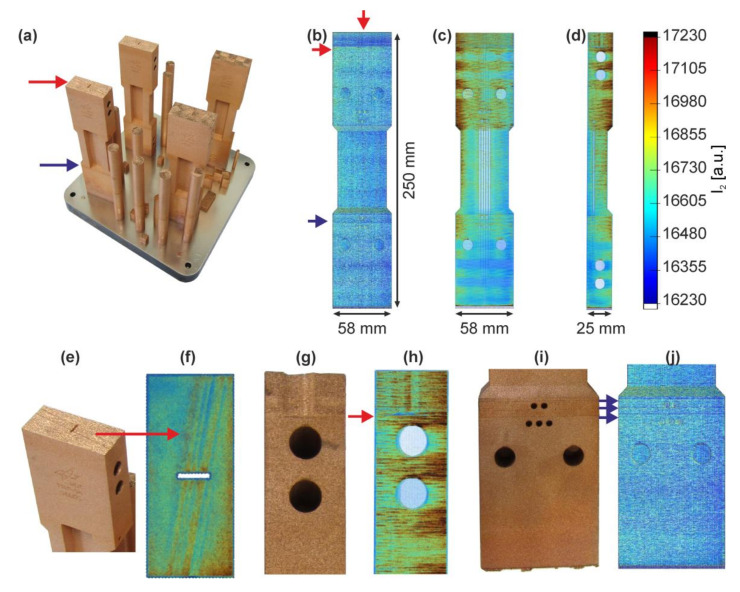
(**a**) The CuCr1Zr-TMF-panel build; (**b**–**d**) MPM data visualization (voxel size of 0.15 × 0.15 × 0.15 mm^3^) of a TMF-panel with 2 different transparency setting; (**b**) the surface view; (**c**,**d**) the visible hotspots and internal structure; (**e**,**f**) the comparison between the visible defects at the top surface and the MPM data (red arrows); (**g**,**h**) the same defect viewed from the panel’s short side shows a defect extending 12.5 mm in height; (**I**,**j**) the lower section of this panel and MPM response shows three distinct horizontal lines (blue arrows).

Individual irregularities in the I_2_ responses were detected on a TMF-panel with full build height (250 mm; Figure 13a), but this time not only limited to the top surface but partially also observed on sides (Figure 13c–e). Different transparency settings for the 3D visualization enable either surface plots (Figure 13b) or the analysis of volume intensities (Figure 13c,d). Again, the spatial distribution of I_2_ mirrors the previous results of the separate segments with higher MPM intensities above diameter reductions.

However, one particular TMF-panel clearly showed several macroscopic defects (locations marked with arrows in Figure 13a) which can be traced back to the MPM I_2_ data recorded during the build (Figure 13b–d). At the top of the specimen, a recoating failure occurred, resulting in several close and parallel vertical lines of ~1 mm width each (Figure 13e,f). Contrary to the surface defect in the cuboid, these defect lines extend several lines below the top layer (≈12.5 mm), showing that coater damage occurred and affected the bulk material in the panel (Figure 13g,h, the vertical feature in this side view). This apparently led to a general decrease in intensity I_2_ measured in this section of the panel (Figure 13b), which is yet to be understood.

Furthermore, the build was paused three times (for machine adjustments to powder sensors) in the lower part of the panel, which caused horizontal lines (“breaks”) at specific build heights that are clearly recognizable in all specimens of the build and marked with arrows on the example in Figure 13i. Again, corresponding lines are visible in the MPM surface plot (Figure 13j). The three pauses were long enough that despite not using any platform heating, the parts cooled down sufficiently to generate a discontinuity on the material upon resumption of the LPBF process.

MPM is clearly suitable for detecting both types of defects, at the surface and within the bulk, which enables identifying failed parts before the build is complete. Parts may be deleted during the build before being finished, allowing time and cost savings. While both types of defects detected here were visible from the part surface itself, it is apparent that such defects can also be detected inside of components. As a comparatively large voxel size of 0.15 × 0.15 × 0.15 mm^3^ was chosen for remapping the MPM data for visualization and analysis, the identification of individual defects was limited only to “macroscopically large” irregularities.

## 4. Conclusions

This study provides new insights into laser powder bed fusion of CuCr1Zr and the usefulness of in situ MPM for the development of process parameters and process understanding as well as for the identification of defects during LPBF of components. The key findings can be summarized as follows:

Two process windows were identified from manufactured cuboids based on the combination of low total porosity, stable processing conditions, and high build-up rates: (i) at t = 30 µm: P = 400 W, 80 µm ≤ h ≤ 100 µm and 400 mm/s ≤ v ≤ 500 mm/s (lower build-up rates) and (ii) at t = 50 µm: P = 400 W, 80 µm ≤ h ≤ 140 µm and 300 mm/s ≤ v ≤ 500 mm/s. Archimedes density measurements, as well as the MPM monitoring, revealed a significant influence of baseplate positioning. One origin for this effect may be attributed to the inert gas flow. The baseplate positioning accounts for deviations of ≈0.5% in total porosity in LPBF, which, besides the typical error bars from the chosen methodology for material density characterization, must also be taken into account, particularly when small porosities are considered.

Importantly, the MPM provided useful guidance for the determination of the process windows: the spatial distribution of the MPM intensities and their variation between subsequent layers (i.e., the standard deviation σ(Ī) of layer-wise averaged signals) allowed us to take into account the process robustness. Although MPM signals integrally averaged over the volume (Î) did not unambiguously correlate with the total porosities (similar to E_V_), in conjunction with E_V_, they allow estimating the total porosity. Thus, MPM can aid in the development of process parameters.

The MPM results obtained from simple shapes like cuboids cannot be transferred directly to complex parts. Different MPM intensity ranges on cuboids and TMF-panels despite similar LPBF parameters show that the MPM intensity distributions depend strongly on geometry and heat transport to the build platform. Analogously, materials properties, such as porosity, evidence the effects of local geometry. Although for cuboids sufficiently dense material was obtained with 400 W laser power, the porosity varied substantially in the TMF-panels manufactured using the same parameters.

MPM allowed for the detection of individual defects and irregularities on the surface and in the bulk of geometrically complex TMF-panels. While these macroscopic defects were also easily visible on the parts later, they can be recognized much earlier in in situ MPM, thus enabling the prediction of defective parts during a build.

## Figures and Tables

**Table 1:** Local porosity results for the sample with the manufacturing parameters t = 30 µm, P = 400 W, v = 300 mm/s, h = 60 µm (compare Figure 9a,b,e,f). For the analysis the LOM, cross-sections were divided into 6 equal areas (see Figure 9f). The total amount of pores, the porosity, and the A_50_ and A_90_ values were calculated as dependent on the different areas for 4 cross-sections. (A_90_: 90% of the 2D-pore areas are smaller than the given value, 10% are larger).

	Area 1	Area 2	Area 3	Area 4	Area 5	Area 6
Pore count	360 ± 45	186 ± 63	410 ± 48	327 ± 8	381 ± 26	381 ± 32
Porosity [%]	0.59 ± 0.18	0.40 ± 0.11	0.61 ± 0.12	0.55 ± 0.03	0.67 ± 0.17	0.58 ± 0.04
A_50_ [µm^2^]	115 ± 18	120 ± 41	119 ± 25	120 ± 25	116 ± 23	117 ± 24
A_90_ [µm^2^]	652 ± 131	838 ± 267	689 ± 105	689 ± 25	677 ± 23	652 ± 24

## Supplementary Materials

The following are available online at https://www.mdpi.com/1996-1944/13/20/4626/s1, Figure S1: Overview images of the selected of LPBF builds. (a) and (b) porosity specimen at t = 30 and 50 µm. (c) build with segments of thermomechanical fatigue (TMF) panels and roughness specimen amongst others, (d) Test build with full TMF panels.2. Surface Quality of Cuboid Specimen Manufactured at t = 30 and t = 50 µm, Figure S2: LOM of surface structures, produced with different process parameters ((a–c) t = 30 µm, v = 300 mm/s, P = 400 W; (d–f) t = 30 µm, h = 120 µm, P = 400 W). The maximum height z_max_ (red) is given on the scale bars, the minimum height in each measurement was set to zero, Figure S3: LOM of surface structures, produced with different process parameters ((a–c) t = 50 µm, v = 300 mm/s, P = 400 W; (d–f) t = 50 µm, h = 120 µm, P = 400 W). The maximum height z_max_ (red) is given on the scale bars, the minimum height in each measurement was set to zero, Figure S4: (a,b) Mean intensity values Ī (specimen process parameters: t = 50 µm, P = 400 W, v = 400 mm/s, h = 60 µm) dependent on the build height with a magnified image of I_2_ in the middle of the specimen. (c) Standard deviation σ(I) of all points in a layer for the intensities I_1_ and I_2_. (d) Mean quotient µ(I_2_/I_1_) for the same specimen.
