# Supplementary material for: Pyrometric-Based Melt Pool Monitoring Study of CuCr1Zr Processed Using L-PBF
Source: Materials (Basel). 2020 Oct 16;13(20):4626. doi: 10.3390/ma13204626 (PMC7603023; doi:10.3390/ma13204626)
Supplement: Supplementary file 1 [file materials-13-04626-s001.pdf]

materials

MDPI

Article

Supplementary Materials: Pyrometric-Based Melt Pool Monitoring Study of CuCr1Zr Processed Using LPBF

Katia Artzt [1,*], Martin Siggel [2], Jan Kleinert [2], Joerg Riccius [3], Guillermo Requena [1,4] and Jan Haubrich [1]

[1] Institute of Materials Research, German Aerospace Center (DLR e.V.; Deutsches Zentrum für Luft-und Raumfahrt), Linder Höhe, D-51147 Cologne, Germany; guillermo.requena@dlr.de (G.R.); jan.haubrich@dlr.de (J.H.)

[2] Institute of Software Technology, German Aerospace Center (DLR e.V.; Deutsches Zentrum für Luft-und Raumfahrt), Linder Höhe, D-51147 Cologne, Germany; martin.siggel@dlr.de (M.S.); jan.kleinert@dlr.de (J.K.)

[3] Institute of Space Propulsion, German Aerospace Center (DLR e.V.; Deutsches Zentrum für Luft-und Raumfahrt), Im Langen Grund, D-74239 Hardthausen, Germany; joerg.riccius@dlr.de

[4] Metallic Structures and Materials Systems for Aerospace Engineering, RWTH Aachen University, D-52062 Aachen, Germany

* Correspondence: katia.artzt@dlr.de; Tel.: +49 2203 601 3293

Received: 31 August 2020; Accepted: 13 October 2020; Published: date

1. LPBF Builds Carried Out for This Study

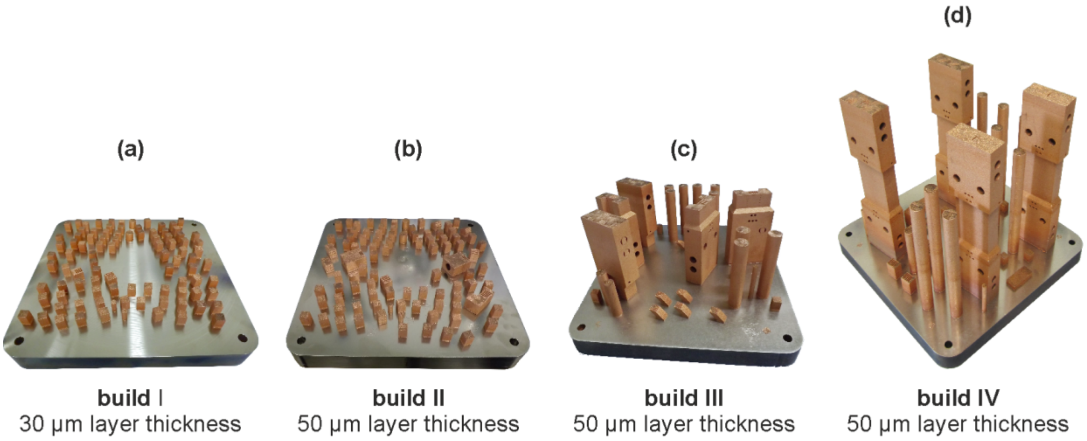

Figure S1. Overview images of the selected of LPBF builds. (**a**) and (**b**) porosity specimen at t = 30 and 50 µm. (**c**) build with segments of thermomechanical fatigue (TMF) panels and roughness specimen amongst others, (**d**) Test build with full TMF panels.2. Surface Quality of Cuboid Specimen Manufactured at t = 30 and t =50 µm.

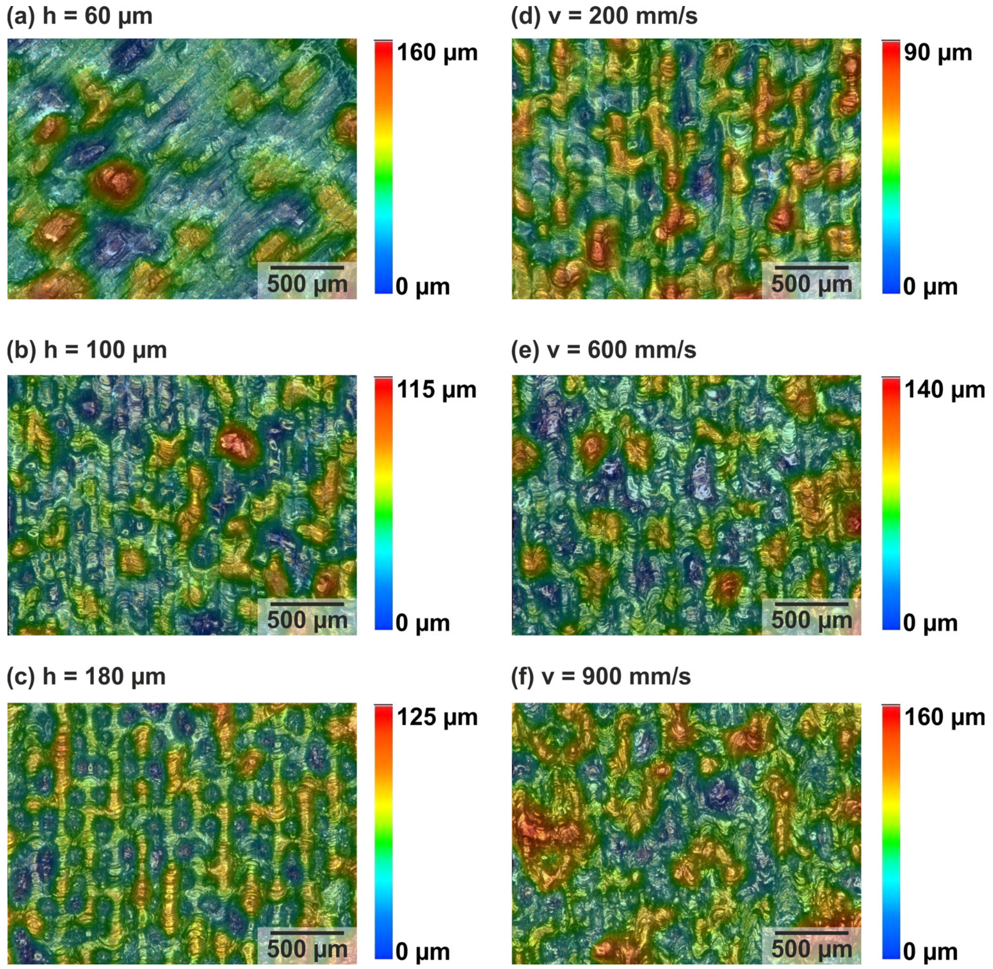

Figure S2. The LOM of surface structures, produced with different process parameters ((**a–c**) t = 30 µm, v = 300 mm/s, P = 400 W; (**d–f**) t = 30 µm, h = 120 µm, P = 400 W). The maximum height z_{max} (red) is given on the scale bars, and the minimum height in each measurement was set to zero.

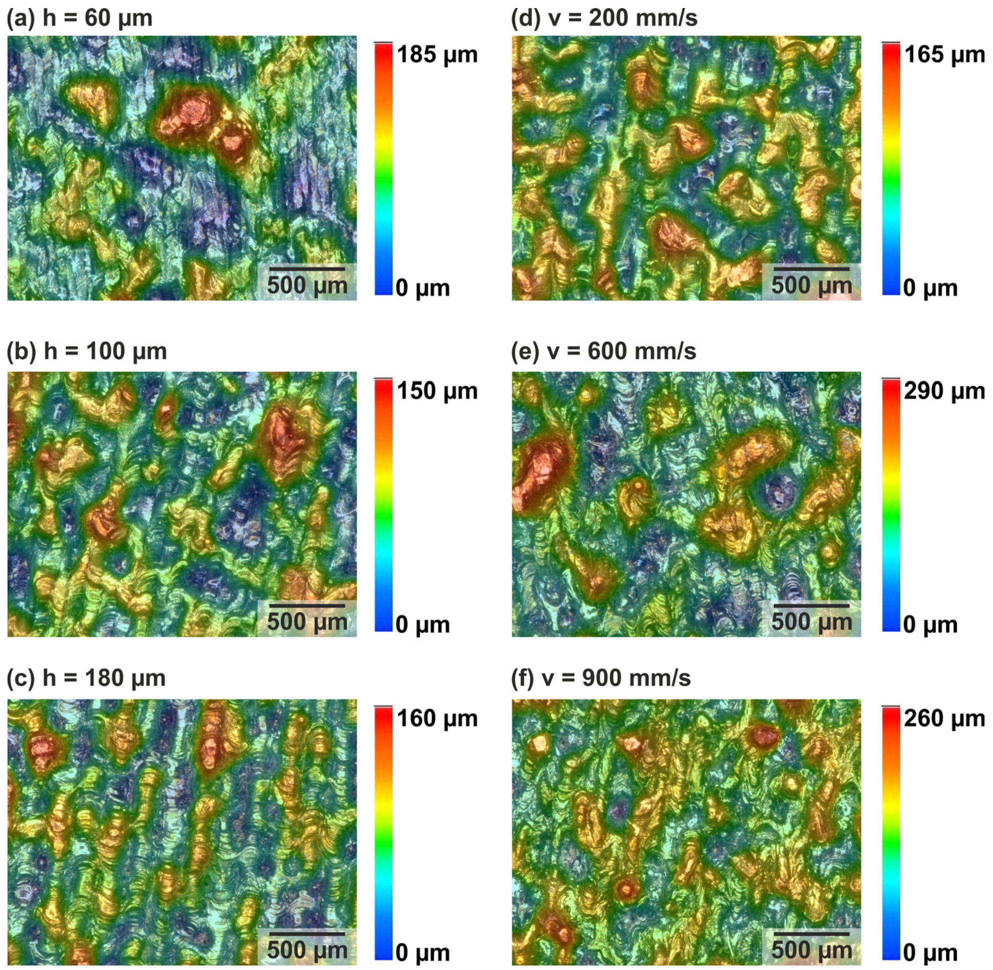

Figure S3. The LOM of surface structures produced with different process parameters ((**a–c**) t = 50 µm, v = 300 mm/s, P = 400 W; (**d–f**) t = 50 µm, h = 120 µm, P = 400 W). The maximum height z_{max} (red) is given on the scale bars, and the minimum height in each measurement was set to zero.

The top surfaces of the small cuboids (10 × 10 × 12 mm^3) manufactured with 114 different process parameter combinations were also analyzed with light microscopy (Figures S2 and S3). No perfectly continuous melt tracks were observed, and the surface of the cuboids is not homogeneous and smooth. The surface structure of CuCr1Zr specimens depends on the process parameters (the example for t = 30 µm is given in Figure S2; for t = 50 µm please see Figure S3, respectively):

For low velocities and small hatch distances (e.g., 300 mm/s and 60 µm), the surface does not show the typical pattern of melt tracks but is dominated by bulges and valleys extending over several melt lines.

With increasing hatch distance and still low velocities (e.g., 300 mm/s and 180 µm), the melt tracks become generally more defined and surface maxima and minima concentrate on single tracks. This apparently represents the most stable melt process condition.

For high velocities and intermediate hatch distances (e.g., 900 mm/s and 120 µm), an inhomogeneous surface structure develops in which small but tall balling features exist next to deep depressions. All surfaces are rough, with maximum heights greater than 150 µm, which again indicates rather unstable process conditions and balling that may favor the formation of pores.

3. Averaging of MPM Intensity Data:

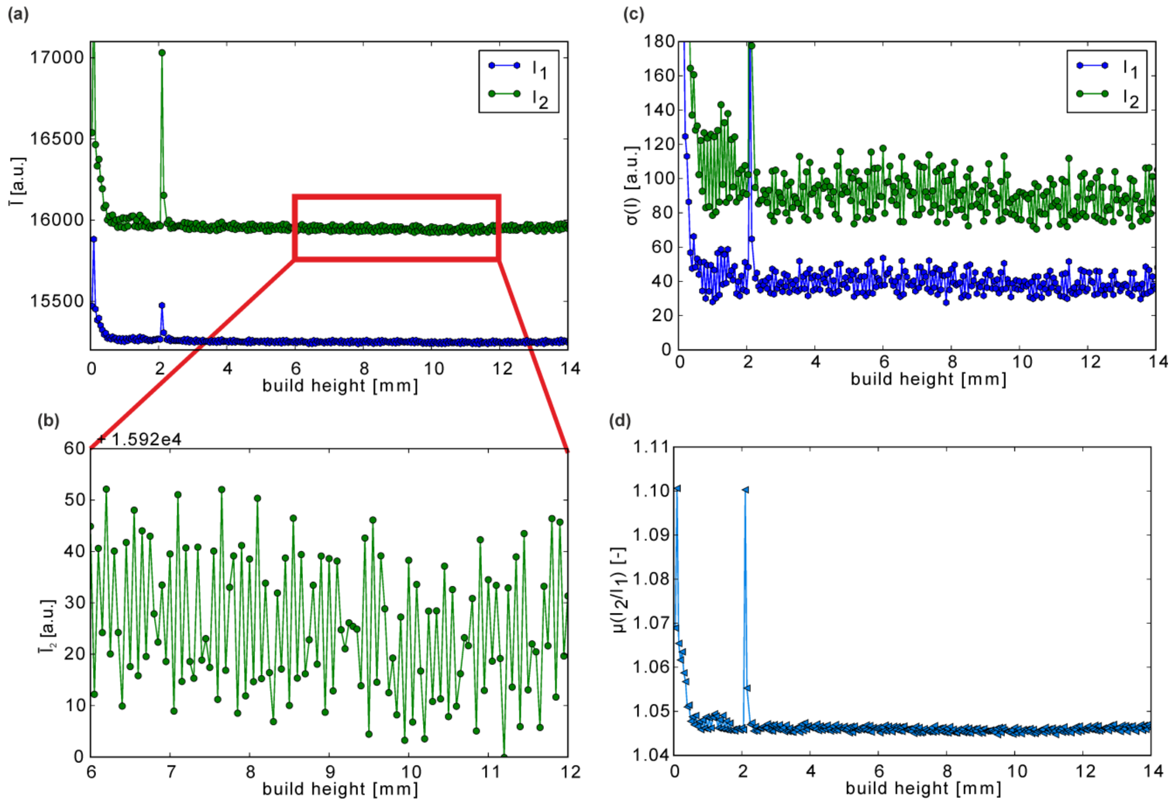

Figure S4. (**a**)–(**b**) The mean intensity values Ī (specimen process parameters: t = 50 µm, P = 400 W, v = 400 mm/s, h = 60 µm) dependent on the build height with a magnified image of I_2 in the middle of the specimen. (**c**) Standard deviation σ(I) of all points in a layer for the intensities I_1 and I_2. (**d**) The mean quotient $\mu(I_2/I_1)$ for the same specimen.

Analogous to the porosity determination by Archimedes' method, mean MPM intensity values for the specimen are computed from the MPM data. For this purpose, first area-mean intensities Ī and area-mean standard deviations (σ) are calculated separately for each layer (and each photodiode). They are presented as a function of the build height (Figure S4) for a build process with a cuboid specimen with:

Two peaks at the build heights b = 0 mm and b = 2 mm are striking in $\mu(I_1)$ and $\mu(I_2)$ (Figure S4a), which are due to:

- increased emission upon forming the first layers on the Ti-6Al-4V base plate at the start of the build job (0 mm) and;
- the transition from the supports to the cuboid bulks (2 mm).

Below this first "massive" layer of the bulk specimen, only thin supports and mostly loose powder particles are present, which strongly reduces heat conductivity (loss) and increases the melt temperature. This results in higher MPM responses.

Besides these two peaks, the mean intensities seem to be constant over the build height within a small scattering band (Figure S4b). Similar effects are also visible in the calculated mean standard deviations per layer (Figure S4c) and the intensity quotient (Figure S4d).

The further analysis of the layer wise mean intensities of the cuboid sections is presented in the main manuscript.
